# Influence of Shape-Forming Elements on Microstructure and Mechanical Properties in Coextruded Thermoplastic Composites

**DOI:** 10.3390/polym17192703

**Published:** 2025-10-08

**Authors:** Rebecca Olanrewaju, Yuefang Jiang, Thao Nguyen, David Kazmer

**Affiliations:** 1 Francis College of Engineering, University of Massachusetts Lowell, Lowell, MA 01854, USA; david_kazmer@uml.edu; 2 Whiting School of Engineering, Johns Hopkins University, Baltimore, MD 21218, USA; yjian106@jhu.edu (Y.J.); vicky.nguyen@jhu.edu (T.N.)

**Keywords:** coextrusion, LCP composites, nylon

## Abstract

The immiscibility of most polymers leads to poor interfacial adhesion in blends, a critical challenge that often limits the mechanical performance of polymer composites. This research introduces shape-forming elements (SFEs), a novel class of coextrusion dies designed to create additional geometric complexity and control over interfacial architecture. Specifically inspired by Julia Set and T-Square fractals, SFEs were simulated, prototyped, and found to be effective in coextrusion of different-colored polymer clays. The SFEs were employed to coextrude architected composites consisting of a liquid crystalline polymer (Vectra A950) and a cycloaliphatic polyamide (Trogamid CX7323). Mechanical testing revealed a strong positive correlation between the draw ratio and both the tensile modulus (adjusted R^2^ = 0.94) and tensile stress at break (adjusted R^2^ = 0.84). However, experimental cross-sections significantly differed from simulation results. These discrepancies were attributed to interfacial instabilities caused by material incompatibility between the two polymers and potential moisture-induced defects. This finding highlights critical challenges that arise during practical processing, emphasizing the importance of addressing polymer compatibility and moisture management to realize the full potential of SFEs in designing advanced polymer composites with targeted properties.

## 1. Introduction

Polymer composites have become increasingly valuable in advanced engineering applications due to their tunable mechanical, thermal, and chemical properties. By combining multiple polymers and fillers, it is possible to leverage the unique attributes of each component. However, a fundamental challenge still remains: most polymer systems are immiscible, leading to phase-separated domains with weak interfacial adhesion. These weak interfaces often limit the mechanical performance of the resulting materials, highlighting the importance of processing techniques that can optimize interfacial properties and enhance overall composite performance [1,2]. To overcome the challenge of immiscibility, various compatibilization strategies are employed to strengthen the interface and refine the blend morphology. Compatibilizers have the ability to interact with both polymer phases to stabilize the interface. Relatedly, reactive compatibilization relies on chemical reactions during processing to form copolymers at the interface [3]. Such approaches have been shown to refine and stabilize the morphology, improve phase adhesion, and enhance the overall performance of polymer blends [1].

The coextrusion of liquid crystalline polymers (LCPs) with nylon presents an area for developing high-performance composite materials, while leveraging the unique properties of both materials. Studies have demonstrated that incorporating LCPs into nylon matrices can significantly improve the tensile strength and modulus. For instance, a composite fiber containing 40% weight LCP showed a 982.1% increase in the tensile modulus and a 123.3% increase in tensile strength compared to pure PA6,6 fibers [1]. This enhancement is attributed to the in situ formation of LCP fibrils during processing, which align along the flow direction, reinforcing the matrix [4,5]. The rheological behavior of LCP/PA blends also plays a crucial role in determining the final properties of the composite. LCPs exhibit low melt viscosity and high shear sensitivity, facilitating their alignment during processing and promoting fibril formation. This alignment is further enhanced during coextrusion, where the shear and elongational flows orient the LCP domains, leading to improved mechanical performance [6]. However, the immiscibility between LCPs and nylon can lead to phase separation and weak interfacial adhesion, potentially compromising the composite’s integrity. To address this, compatibilization strategies, such as the addition of adhesion-promoting polymers or reactive compatibilizers, have been employed to enhance interfacial bonding [7]. Furthermore, the processing conditions during coextrusion, including the temperature, shear rate, and draw ratio, significantly influence the morphology and properties of the resulting fibers. Optimizing these parameters is essential to achieve the desired alignment and dispersion of LCP fibrils within the PA matrix [8]. Advanced coextrusion techniques, such as multilayer coextrusion, have also been explored to fabricate composites with tailored architectures, offering more precise control over layer thickness and composition [9,10].

Agassant and Demay provide a comprehensive review of the polymer coextrusion processes, with a focus on the modeling of multilayer flows and the critical challenges associated with interfacial stability. The authors developed one-dimensional (1D) bilayer coextrusion models for both Newtonian and shear-thinning fluids, demonstrating that stationary interface positions and velocity profiles are established after a short distance, justifying the use of 1D stationary models for certain conditions. A significant portion of the review addresses interfacial instabilities that can arise during coextrusion. The authors note that such instabilities may be observed depending on the rheological properties of the coextruded polymers and their flow rate ratios. These instabilities can be amplified along the die land, leading to defects in the final product. Convective stability analysis and direct numerical computations are employed to distinguish flow situations that amplify or dampen these instabilities [11].

Jape and Deshpande conducted a comprehensive study on the morphology, crystallization, and melting behavior of melt mixed blends of nylon 6,6 (PA6,6) and a thermotropic liquid crystalline polymer (LCP), specifically Vectra A950. The research aimed to understand how varying concentrations of LCP influence the thermal and morphological properties of the PA6,6 matrix. The study revealed that the PA6,6 and LCP blends are immiscible across all compositions, leading to distinct phase separation. Despite the immiscibility, the inclusion of LCP, particularly at lower concentrations, was found to accelerate the crystallization rate of the PA 6,6 matrix. This enhancement is attributed to the nucleating effect of the LCP, which facilitates the formation of smaller crystallites in the growth direction. Thermal analysis conducted indicated a decrease in the equilibrium melting temperature of the blends compared to pure PA6,6. The addition of LCP within the matrix also influenced the melting behavior [12].

Layer-multiplying elements (LMEs) have emerged as a significant advancement in coextrusion technology, enabling the fabrication of multilayer polymer films with controlled architectures. By repeatedly splitting and stacking polymer streams, LMEs can produce films with hundreds to thousands of alternating layers, each potentially as thin as a few nanometers [2]. The multilayer structure can lead to improved mechanical, barrier, and optical properties due to the increased interfacial area [13]. Recent work in multilayer coextrusion has shown that such rheological contrasts can disrupt flow uniformity, but also that increasing the interfacial density can significantly enhance processing stability and strain hardening of the composite as a whole [14]. Building on computational modeling of shape-forming elements, this research investigates a new approach for tailoring polymer composite architectures: the usage of shape-forming elements (SFEs) during coextrusion. Unlike layer-multiplying elements (LMEs), SFEs are intended to enable precise control across the composite’s 2D cross-section [15]. This potential capability opens up new possibilities for manipulating molecular alignment and anisotropy, particularly in blends containing LCPs, which are known for their fibrillar anisotropy, to further enhance mechanical properties [16,17]. LCPs are a class of polymers that exhibit a unique combination of high strength, stiffness, and thermal stability due to their ordered molecular structures. When incorporated into polymer blends, LCPs can form fibrillar structures that reinforce the matrix, leading to composites with superior mechanical properties. The alignment of LCP domains during processing is important, as it influences the degree of reinforcement and anisotropy in the final material [18,19,20,21].

This study aims to design and fabricate coextruded architected composites using an amorphous polyamide (APA) as the matrix and LCP as the reinforcement. The specific objectives of this research are as follows: to evaluate the impact of shape-forming elements and the polymers used on the resulting cross-sectional architecture, to assess the mechanical properties of the composites as a function of SFE-induced architecture, and to investigate the influence of the draw ratio on molecular alignment and anisotropy. By transitioning from the one-dimensional control of LMEs to the two-dimensional control offered by SFEs, this study explores a more versatile method for optimizing structure–property relationships in LCP-based polymer composites.

## 2. Materials and Methods

### 2.1. Shape-Forming Elements

**Figure 1:**
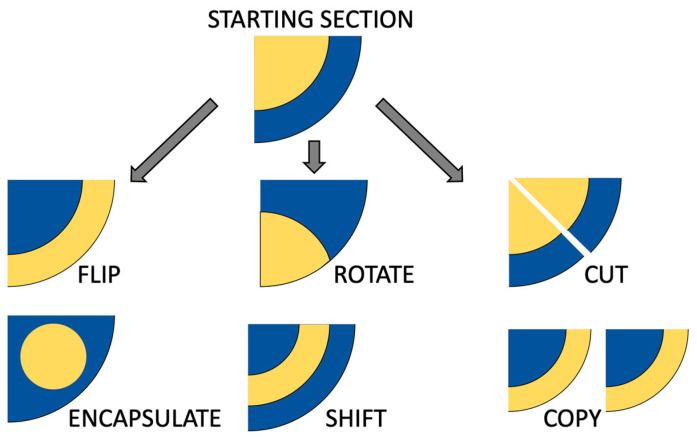
Outline of potential operations of SFEs, where the starting section is a core–shell configuration of coextruded flow, and the potential resultant cross-sections.

In an abstract sense, shape-forming elements (SFEs) represent a new class of modular coextrusion dies, capable of performing additional manipulation of multilayer polymer structures. SFEs can execute a wide range of operations, each contributing to the controlled architecture of the final composite. For example, conceptual operations that SFEs can conduct with the coextruded material include flipping the material arrangement, which essentially would invert material placement. Another potential operation is rotating the material flow, which reorients the materials and shifts them axially. Moreover, SFEs can also be designed to cut the extrudate, introducing controlled interruptions or breaks in the sample. The encapsulation operation, where one material fully surrounds another, is another possibility that enables the creation of additional core–shell structures. SFEs can also shift a portion of material within the die, moving a particular architecture to another area. Finally, copying operations would allow SFEs to duplicate existing material arrangements, effectively multiplying the local architecture without introducing additional extruders, a concept similar to LMEs but extended to more complex geometries. Unlike conventional LMEs, which multiply and stack thin layers in a uniform manner, SFEs can create non-uniform, position-dependent architectures, making them particularly attractive for producing anisotropic composites with tailored properties. The flexibility of SFEs would make them an ideal tool for exploring novel design spaces in coextruded composites, especially those that involve immiscible materials like PA and LCP, where the control of phase morphology and interfacial structure is paramount. These conceptual SFE operations are illustrated in Figure 1.

A key objective of utilizing SFEs in coextrusion is to maximize the perimeter-to-area ratio of the resulting polymer composite cross-section. By introducing more intricate geometries such as branching, finger-like protrusions, or finely segmented channels, the SFEs could effectively extend the interfaces between the constituent polymers while maintaining a relatively constant cross-sectional area of each phase. This design strategy promotes more mechanical interlocking between the typically immiscible phases. The increased perimeter-to-area ratio, by maximizing interfacial contact between the coextruded materials, allows the composite to develop strong physical interlocking at the interface, eliminating the reliance on chemical compatibilizers that are often used to promote interfacial adhesion in polymer blends.

**Figure 2:**
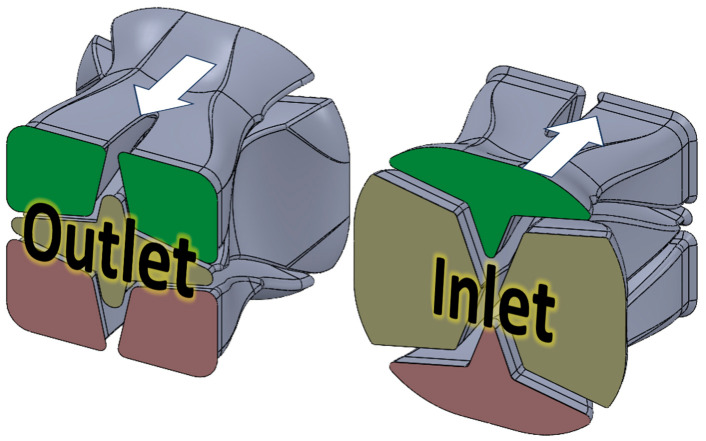
Chevron SFE flow path geometry.

For this study, two different SFEs were designed and evaluated: (1) the Chevron and (2) the Input–Output (I.O.). The details of the Chevron flow path are illustrated in Figure 2. The main goal of the Chevron design is to cut the center of the extrudate, highlighted in gold at the inlet location in Figure 2, into left and right sections, and to push the top and bottom corner sets, green and pink, toward the middle of the flow path.

**Figure 3:**
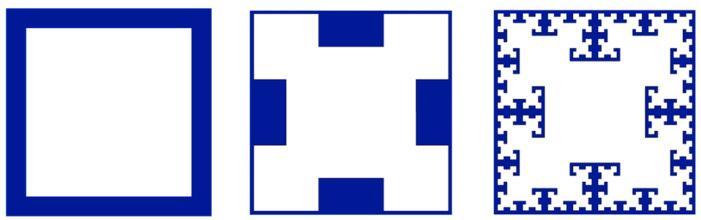
The progression of the T-Square fractal with added complexity from left to right.

The aim of the Chevron design is to create the T-Square fractal, shown in Figure 3, when several SFEs are used in a stacked configuration. The T-Square fractal has high interfacial area as well as interlocking regions between the two materials compared to traditional composite designs [22].

**Figure 4:**
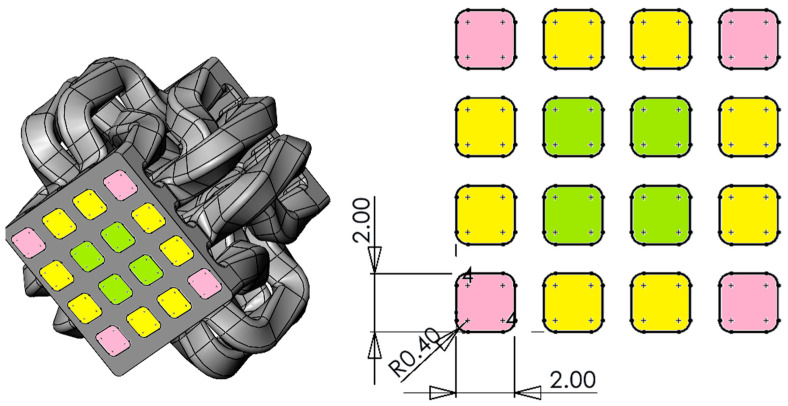
Input–Output (I.O.) SFE flow path geometry.

The second SFE, named Input–Output (I.O.), in Figure 4, has a digital material control approach compared to the Chevron design, with a 4 × 4 grid of ports at both the inlet and the outlet. The side ports, shown in yellow, are rotated 90 degrees while material is flowing through. The corner ports and center ports, marked as pink and green, swap places at the end of the SFE, while also rotating 180°.

### 2.2. Materials

**Figure 5:**
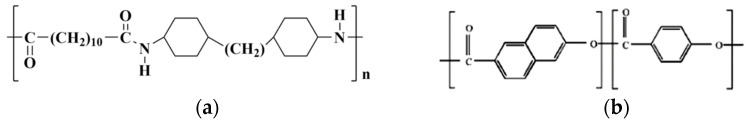
(**a**) Trogamid CX7323 monomer [24]; (**b**) Vectra A950 monomer.

The material system was coextruded liquid crystalline polymer acting as a reinforcing fiber in a cycloaliphatic polyamide matrix (Trogamid CX7323, Evonik Industries, Piscataway, NJ, USA in Figure 5). Trogamid CX7323 is a microcrystalline transparent polyamide, with crystallites small enough that they do not scatter visible light, appearing transparent [23]. The Trogamid CX7323 monomer consists of a dodecanedioic acid and saturated cycloaliphatic ring units between the amide linkages [24]. The rings present in the monomer give rigidity to the polymer backbone, enhance chemical resistance, and minimize crystallization. The cycloaliphatic diamines have a low refractive index, contributing to the transparency of the polymer. The long, flexible dodecanedioic acid provides additional flexibility within the monomer, enhancing its toughness [24,25,26].

Table 1 shows a compilation of the polymers’ respective mechanical properties. Coextruding these materials with the SFE designs allows for strategic placement of each polymer (and its properties) within the cross-section. Using these materials could offer several distinct benefits. Vectra A950 (Celanese, Irving, TX, USA), with a tensile modulus of 7800 MPa and a yield stress of 148 MPa, provides high stiffness and strength. Conversely, Trogamid CX7323, with a lower tensile modulus of 1400 MPa and a higher yield strain of 8%, introduces toughness and flexibility into the material system while reducing brittleness. These properties make the coextrusion of LCP and APA an ideal approach to create composites with synergistic properties.

### 2.3. Rheology

#### 2.3.1. Polymer Clay

Polymer clay was used as a physical modeling material to prototype and visualize flow through the SFEs prior to full-scale polymer coextrusion. Polymer clay served as a convenient analog for capturing deformation through the SFEs due to its ease of handling and pseudoplastic behavior at room temperature. The rheological properties of the polymer clay were characterized and fitted to a model suitable for computational fluid dynamics (CFD) implementation.

Capillary rheometry was conducted at ambient temperatures using two dies with L/D ratios of 5 and 15 (Die IDs CX394-05, CX39415) via a Dynisco LCR 7000 (Franklin, MA, USA) capillary rheometer. The tests covered a low-to-moderate shear rate range (200 to 1 s^−1^), matching the conditions experienced by the clay during slow, piston-driven extrusion in the prototyping trials. No entrance correction (e.g., Bagley) or non-Newtonian correction (e.g., Rabinowitsch) was applied; instead, the apparent shear viscosity values from the raw data were directly imported into ANSYS Polyflow 2022 R2. Within Polyflow, the material’s viscosity profile was automatically fitted using the Bird–Carreau–Yasuda (BCY) model:

(1) $$\eta \left(\dot{\gamma }\right)=\eta_{\infty }+\left(\eta_{0}-\eta_{\infty }\right){\left[1+{\left(\lambda \dot{\gamma }\right)}^{a}\right]}^{\frac{n-1}{a}}$$

where $\eta_{0}$ and $\eta_{\infty }$ are the zero- and infinite-shear viscosities, λ is the critical time constant for shear-thinning onset, n is the power-law index at high shear rates, and a is a shape parameter that controls the curvature of the transition region. This model was selected because it captures both Newtonian plateaus and the gradual onset of shear thinning with greater fidelity than simpler three-parameter models like Carreau or Cross. Its mathematical form ensures numerical smoothness and stability with finite-volume and finite-element solvers, making it well suited for simulating flows of high-viscosity pseudoplastic materials like polymer clay.

In the range tested, the clay exhibited pseudoplastic behavior, with a distinct Newtonian plateau at very low shear rates, followed by a sharp reduction in viscosity as the shear rate increased. This trend is consistent with viscoplastic modeling materials, in which internal structure resists deformation at rest but breaks down readily under applied shear [27]. To minimize the influence of entrance effects and obtain a more accurate estimate of the true shear viscosity, only data from the longer die (L/D = 15) were used for fitting the BCY model and for subsequent simulations. The longer die length ensures that the flow is fully developed over a greater distance, reducing the influence of entrance effects and providing a more accurate estimate of true shear behavior. The 5 L/D die, by contrast, includes a significant proportion of pressure drop due to entrance losses, particularly at low shear rates, leading to inflated apparent viscosity values [28,29]. The resulting fitted rheological model was used for the simulation of polymer clay flow through the same SFE geometries used in physical prototyping and polymer processing.

#### 2.3.2. Polymers

**Figure A2:**
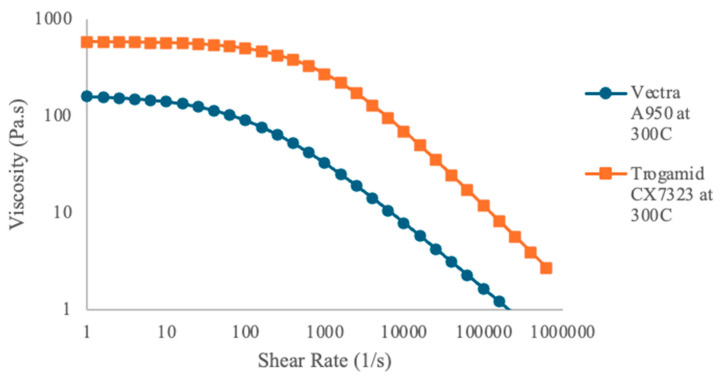
Fitted rheological comparison of Vectra A950 and Trogamid CX7323.

The viscosities of the APA and LCP grades are shown in Figure A2, fitted with the Cross–WLF model. The corresponding Cross–WLF model coefficients are exhibited in Table A3, which were obtained from the Autodesk Moldflow database, including experimentally measured viscosity values across a range of temperatures and shear rates representative of typical polymer processing conditions. Both materials are displayed for visual comparison of the differences in their rheological properties, at 300 °C. This model combines the Cross equation for shear-thinning behavior with the Williams–Landel–Ferry (WLF) model to describe the temperature dependence of polymer viscosity:

(2) $$\eta \left(\dot{\gamma },T\right)=\frac{\eta_{0}\left(T\right)}{1+{\left(\frac{\eta_{0}\left(T\right)\dot{\gamma }}{\tau^{*}}\right)}^{1-n}}$$

where

(3) $$\log_{10}\left(\eta_{0}\left(T\right)\right)=D_{1}+\frac{D_{2}}{T-T_{ref}+D_{3}}$$

In this formulation, $\eta \left(\dot{\gamma },T\right)$ is the apparent viscosity at a given shear rate and temperature, $\eta_{0}\left(T\right)$ is the temperature-dependent zero-shear viscosity, $\tau^{*}$ is the critical shear stress at the onset of shear thinning, and n is the power-law index defining the degree of pseudoplasticity. The constants $D_{1},D_{2},$ and $D_{3}$ originate from the WLF equation and capture how viscosity shifts with temperature. This model structure is widely accepted in the polymer processing literature and simulation software due to its ability to represent real polymer melt behavior over large temperature and deformation ranges [30,31,32,33]. The resulting model was directly applied in ANSYS Polyflow to simulate coextrusion through the shape-forming elements.

Both polymers exhibit shear-thinning behavior, where their viscosities decrease as the shear rate increases. However, Vectra A950 displays a more pronounced shear-thinning effect. Specifically, Vectra A950′s viscosity drops significantly from approximately 100 Pa-s at low shear rates to below 10 Pa-s at higher shear rates. In contrast, Trogamid CX7323 maintains a significantly higher viscosity across the entire shear rate range, decreasing more gradually from approximately 1000 Pa-s and remaining more viscous than the LCP even at high shear rates. The higher initial viscosity of the APA suggests that the molecular structures of the materials have a significant effect on the viscosity differences. LCPs typically experience more molecular alignment under shear, leading to a rapid reduction in viscosity at elevated shear rates. When Vectra A950 and Trogamid CX7323 are coextruded at the same temperature, the significant differences in their viscosity profiles can cause processing challenges. This viscosity mismatch can result in flow instabilities and pressure imbalances in the die, which can cause issues like die swell, voids, and an irregular flow profile.

### 2.4. Simulation

#### 2.4.1. Polymer Clay

ANSYS Polyflow simulations were conducted using the rheological data for polymer clay to model its flow behavior through the Chevron and I.O. SFE flow path geometries. This modeling effort aimed to replicate and predict the behavior of the polymer clay during physical extrusion trials, enabling a direct comparison between simulated flow patterns and clay extrudates. For these simulations, all settings and boundary conditions were kept consistent across both Chevron and I.O. designs, as summarized in Table A2. The simulation type selected was the Generalized Newtonian Isothermal Flow problem, in which viscosity is dependent primarily on shear rate, and the temperature effects within the flow system are negligible. The assumption is appropriate for polymer clay, which was extruded at room temperature and exhibits negligible thermal variation during the process due to the low shear rates experienced by the material.

The fitted BCY model was used in ANSYS Polyflow to define the clay’s shear-dependent viscosity. In addition, several standard assumptions were applied in the simulation: a no-slip wall boundary condition, steady-state flow, and an even core/shell material distribution during coextrusion. Although only a single polymer clay material was used, the coextrusion condition was still applied in the simulation. This was achieved by creating two inlet domains, each assigned the same rheological properties as the polymer clay, to simulate the flow of two distinct streams, representing the different clay colors used in the prototyping process. This setup mirrored the physical experiment, where dual-colored clay was used to visualize the interface evolution and assess the deformation induced by the SFE geometries.

#### 2.4.2. Polymers

**Figure 6:**
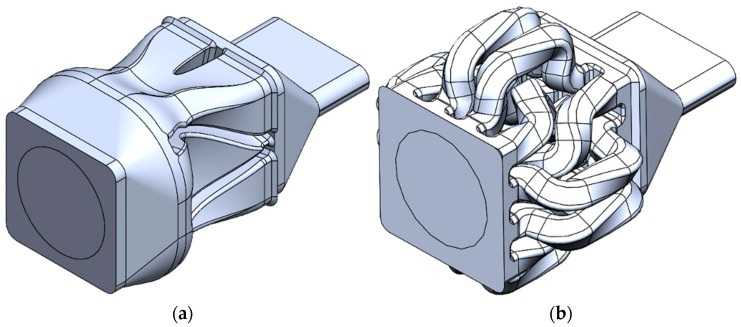
Flow paths for (**a**) Chevron and (**b**) I.O. designs with the slot die geometry incorporated.

ANSYS Polyflow simulations were run on both SFE configurations, Chevron and I.O., to evaluate the ability of the SFEs to create complex cross-sections. The geometry modeled in this full 3D simulation included the starting coextrusion condition and SFE flow path and slot die geometry, shown in Figure 6. The starting coextrusion configuration follows the assumption that the material is completely centered, with the area fractions of the coextruded material equaling 50/50.

For this simulation portion, all settings used for both Chevron and I.O. simulations are outlined in Table A4. The problem type utilized for both simulations was defined as the Generalized Newtonian Isothermal Flow problem, where the material viscosity is dependent on the shear rate more than the temperature, which is suited for simulating the flow of polymers, where the viscosity is shear-rate-dependent. With this problem type, the assumption is that temperature changes within the flow system are negligible, simplifying the problem. The rheological model used for the simulations was the Cross model, chosen for its ability to model both Newtonian and shear-thinning behaviors present in polymers. Within the simulation, several assumptions were made, such as the no-slip wall condition and the even core/shell distribution during coextrusion.

### 2.5. Prototyping

#### Polymer Clay

**Figure 7:**
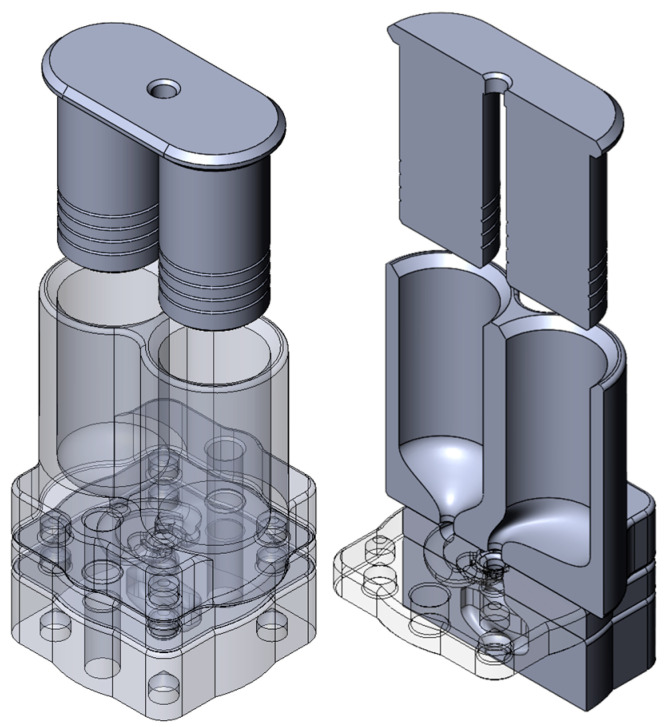
Three-dimensional model of piston-driven polymer clay extruder for SFE validation.

For concept validation of shape-forming elements (SFEs), a 3D-printed extruder prototype, outlined in Figure 7, was utilized. This prototype is a piston-driven system with separate chambers for the two materials and a coextrusion feed manifold. The extruder prototype was 3D-printed via a Stratasys F370 FDM printer with PC-ABS (Item no. 333-6700) and soluble QSR soluble support material (Item no. 333-63500). Subsequently, the shape-forming elements can be stacked or rotated axially to change the material flow operations. Two different colors of Crayola Dough polymer clay were utilized to observe where the material starts and ends. The internal portion, or core, of the concentric circles’ arrangement utilized yellow polymer clay (Item no. 5700153034), and the external portion, or shell, of the concentric circles’ arrangement utilized blue polymer clay (Item no. 5700153042).

### 2.6. Extrusion Processing

#### Polymers

**Figure 8:**
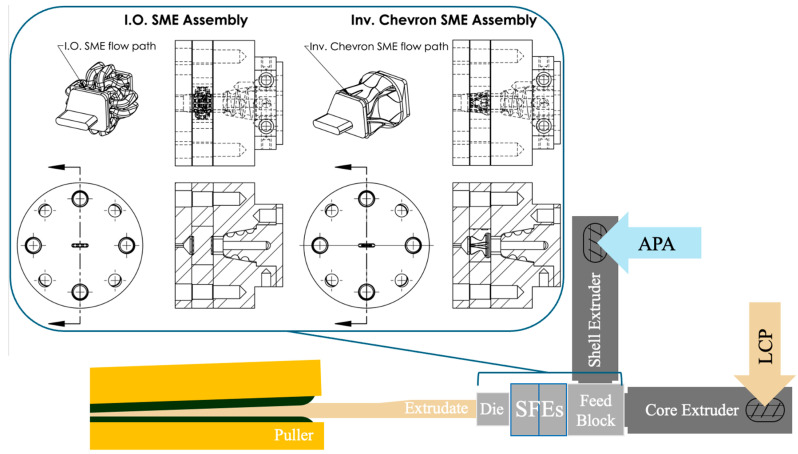
Processing setup for both SFEs utilized, showing the flow of material from each extruder, through the feedblock, through the SFEs and slot die, and to the puller.

The coextrusion process was conducted using a coextruder comprising two Jugetek (Shanghai, China) single-screw extruders (screw length and diameter of 225 and 20 mm, respectively), each having three-barrel temperature zones and screw speed control. The two melt streams are brought together in a custom-designed spiral flow manifold (Figure 8), which directs the core material straight through the center and the shell material in a spiral path, encasing the core. In this setup, the core polymer flows through the center of the manifold and exits straight into the die, while the shell polymer flows along a spiral pathway that wraps around and encases the core stream. The result is a coextruded extrudate providing two materials in a core/shell configuration feeding into the die. The feed throat for each extruder was fit to a custom hopper that receives a thread 473 mL glass jar containing the feedstock to ensure that as little moisture enters the extruder as possible. The materials, Vectra A950, used as the core material, and Trogamid CX7323, used as the shell material, were fed into the extrusion process using the starve-fed method. The materials were dried prior to extrusion at 150 °C for 12 h to eliminate as many moisture-induced defects as possible, including bubbles and voids, which create stress concentrations within the sample and compromise the resultant mechanical properties. All coextrusion experiments were conducted in ambient air, with the process allowed to stabilize for 10 min before collecting samples. Each extrusion run lasted for 45 min, providing sufficient material for subsequent mechanical testing and microscopy. Table 2 highlights the process settings used during coextrusion for both the Chevron and I.O. SFEs.

### 2.7. Microscopy

#### Polymers

Microscopy was employed to examine the resulting cross-sections and material distribution within the samples after the coextrusion process and passage through the SFEs so as to characterize the material distribution among the samples’ cross-sections as well as the fracture behaviors caused by varying SFE configurations. To achieve this, cross-sectional images were captured using a Zeiss Discovery V20 (Oberkochen, Baden-Württemberg, Germany) optical stereo microscope. To prepare the samples for microscopy, sections transverse to the flow direction were made using a Buehler Isomet 1000 precision saw (Lake Bluff, IL, USA) equipped with a diamond blade, ensuring clean, undistorted, and polished surfaces for observation. Observations were conducted under ambient conditions with a dark background, which enhanced the visibility of microstructural details. In addition to evaluating the samples at the cross-section transverse to flow, the imaging was also performed with the fracture surfaces of the tensile-tested samples. This imaging allowed for the correlation of microstructural characteristics, such as material distribution and the draw ratio, with the mechanical properties of the samples.

### 2.8. Structural

#### Polymers

Tensile testing was performed on all SFE configurations using an Instron 34SC-2 universal testing machine, which was equipped with a 3 kN load cell and mechanical grips. The tests were conducted in accordance with ASTM D638 [34], with a crosshead speed of 5 mm/min and a gauge length of 50 mm, corresponding to an initial strain rate of 0.17%/s. Specimens were tested as extruded strips, prepared directly from the coextrusion process. Samples were stored at room temperature prior to testing and were not dried, and the tests were carried out at 21 °C. No extensometer was used, and strain was calculated based on crosshead displacement. The grip dimensions were standardized at 19 mm by 19 mm to ensure uniformity across all samples. For each SFE configuration, five specimens were tested, and results are reported as mean values without outlier removal.

The primary goal of these tests was to evaluate the mechanical properties of the materials, including the modulus, stress at break, and strain at break, and to establish correlations between these properties, the draw ratio, and the specific SFE configuration employed during processing. The elastic modulus was determined as the slope of the initial linear region of the engineering stress–strain curve, while stress at break was defined as the engineering stress at the fracture load, and strain at break was taken as the engineering strain at the point of fracture. All values are reported in terms of engineering stress and strain, as calculated automatically by Instron Bluehill software (v4.55).

Beyond characterizing the mechanical properties, tensile testing also provided insights into the influence of LCP, the specific SFE used, and the draw ratio on the samples’ break behavior and material distribution. The testing outcomes were important in understanding how the differences in SFE geometry impact the structural integrity and performance of the samples under tensile loads. By combining the mechanical property data with microscopy results, the results helped with gaining further understanding of the influence of the SFE on material distribution and mechanical properties.

## 3. Results and Discussion

### 3.1. Simulation

#### 3.1.1. Polymer Clay

**Figure 9:**
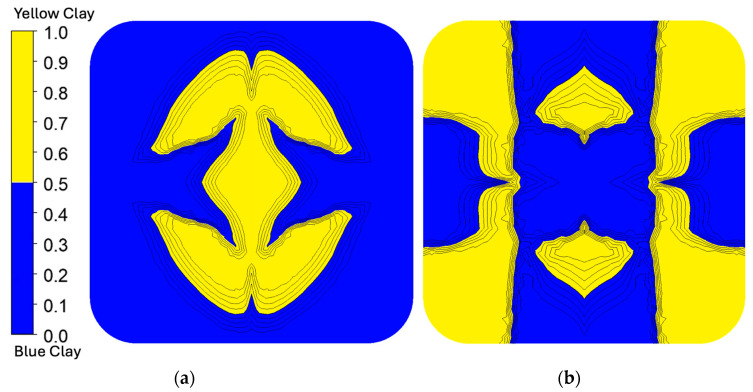
Polymer clay fluid fraction results for (**a**) Chevron and (**b**) I.O. SFEs. In the Chevron SFE, the yellow clay remains centralized, consistent with low shear through the core of the die. In contrast, the I.O. SFE produced a more complex deformation, with the yellow stream diverging laterally and partially wrapping around the central flow.

The simulation results for polymer clay extrusion, shown in Figure 9, illustrate the flow redistribution within the Chevron and I.O. SFEs under simplified coextrusion conditions. To simulate the coextrusion of two-colored materials, the inlet flow domain was divided into two regions of identical rheology to mimic the coextrusion of the dual-colored clay streams. This configuration allowed for the observation of interface evolution and deformation within the different SFEs, with the absence of the material property contrast that is present in the true LCP/APA system.

In the Chevron SFE, the polymer clay simulation predicted a largely centralized distribution of the yellow stream, where the behavior is consistent with the low shear expected through the center of the flow path, where the low-viscosity LCP from the polymer simulation also maintained its position in the core. The I.O. configuration, however, revealed a more complex deformation pattern. In the polymer clay simulation, the yellow stream diverged from the center and formed lateral lobes that wrapped partially around the central flow, with isolated blue regions persisting near the middle of the slot die. Several discrepancies should be noted.

#### 3.1.2. Polymers

**Figure 10:**
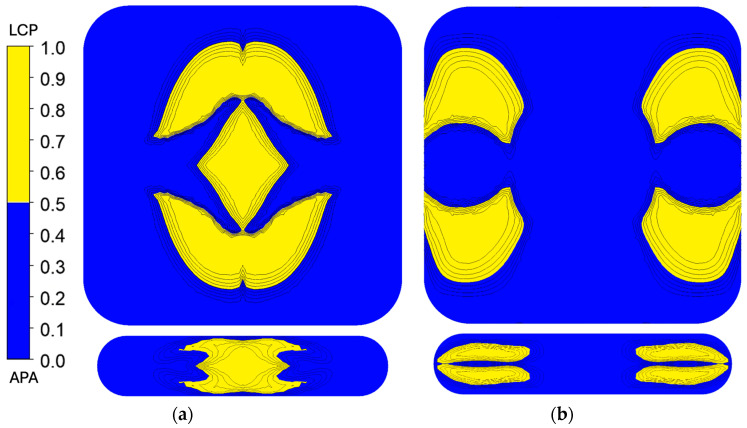
LCP fluid fraction results for Chevron (**a**) and I.O. (**b**). In both geometries, the low-viscosity LCP was predicted to remain in the core, while the higher-viscosity APA occupied regions closer to the die walls.

The simulation results, shown in Figure 10, provide insights into the flow behaviors and rheological behavior of the LCP and APA during coextrusion. APA, being highly viscous, resists deformation under shear and tends to dominate the regions near the die walls, where flow resistance is higher. LCP, with its lower viscosity, flows more easily and occupies the core regions of the extrudate. While the simulation assumes consistent behavior at 300 °C, real-world factors, such as localized temperature variations, shear heating, and material interactions at the interfaces and at the die walls, can often lead to deviations in results. The APA is more likely to experience slight drops in viscosity near the walls due to shear heating, allowing more LCP intrusion than predicted. Similarly, any imperfections in die surface finish or geometry, material incompatibilities, moisture, or other minor thermal degradation during extrusion could contribute to uneven material distribution, creating transitional zones not captured in simulation. The simulation predicted that the low-viscosity LCP would remain largely in the core, while the high-viscosity APA would occupy the regions near the die wall for both SFE geometries.

In the Chevron SFE design, the diamond shape in the flow path introduces sharp transitions and high shear gradients, significantly influencing the material placement. The LCP remains largely centralized due to its low viscosity, maintaining its position within the core region that was established by the initial core–shell geometry. The APA, resisting shear deformation, stays primarily near the die walls, consistent with its high-viscosity characteristics. The experimental results can reveal slight deviations from this behavior, such as uneven interfaces or loss of geometry within the SFE. Simulations, which assume smooth flow transitions and uniform material properties, may overemphasize the uniformity of LCP placement and underpredict these boundary-layer disruptions.

For the I.O. configuration, the core–shell geometry also remains a strong determinant of material placement. The higher viscosity of the APA ensures that it predominantly occupies the wall regions, but experimental results could suggest some intrusion of LCP into these zones, likely due to the combination of flow dynamics and localized shear effects, which may not have been completely captured by simulation. The polymer clay extrusion and microscopy indicate that the material behavior allowed for greater material redistribution than predicted. Additionally, thermal effects, such as shear heating, might further facilitate LCP intrusion in an experimental setup, creating less distinct boundaries than those shown in the simulation results.

The polymer clay results revealed some discrepancies compared to expectations derived from simulation. For the Chevron SFE, the redistribution of the core matched closely, whereas the I.O. SFE polymer clay prototype highlighted the challenges of matching the polymer clay and simulation results.

### 3.2. Prototyping

#### Polymer Clay

**Figure 11:**
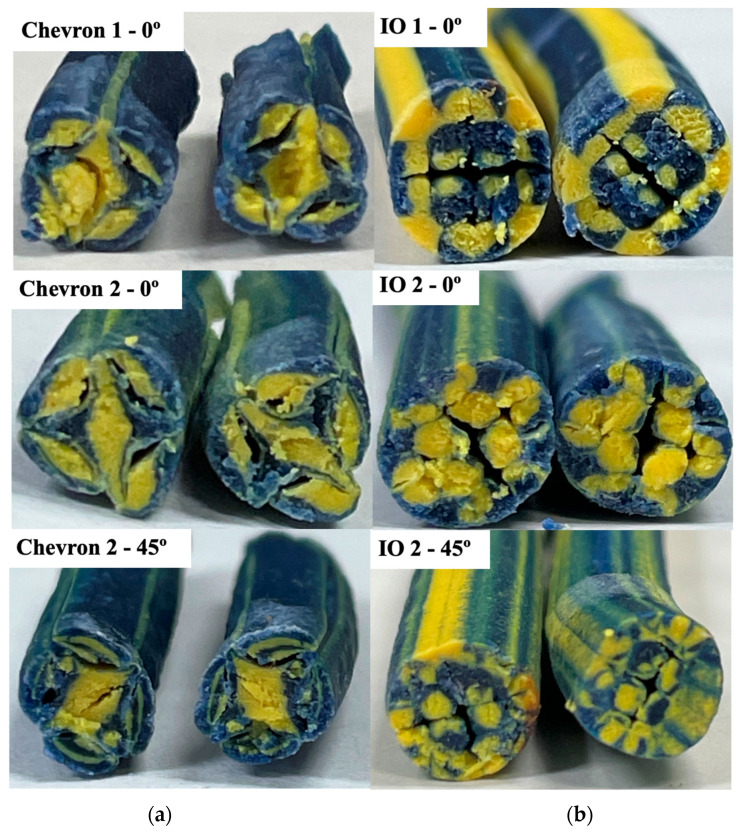
Results of polymer clay validation for Chevron (**a**) and I.O. (**b**). For single-SFE runs, the Chevron largely preserved the initial core–shell structure, while the I.O. showed greater material redistribution. Stacked and rotated configurations increased interfacial area and mixing, with the I.O. producing more pronounced deformation and intermingling of core and shell materials compared to the Chevron.

The resulting cross-sections utilizing the polymer clay and piston-driven extruder system are illustrated in Figure 11. Each SFE run had several different configurations: one SFE, two SFEs stacked, and two SFEs stacked with one rotated axially 45° from the other. The resulting cross-sections demonstrated distinct patterns of material distribution influenced by both the SFE configurations and starting configurations. For the single-SFE setup, the core–shell structure from the initial concentric configuration was largely maintained for the Chevron SFE, while the I.O. SFE showed more distribution. However, localized distortion occurred near regions of sharp transitions in the flow path, where the shell material (blue clay) intruded slightly into the core region (yellow clay). This observation suggests that even simple SFE configurations can induce mild redistribution of materials, particularly in high-shear zones. In the stacked SFE configurations, the Chevron SFE exhibited less extensive redistribution of materials. Although the Chevron SFEs were stacked, the core material remained predominantly at the center. The initial cutting action effectively separated the core material into some distinct sections, while the outward displacement of these sections brought the shell material into closer proximity with the core. This increased interaction between the two materials at the interfaces suggests potential mixing or blending effects, especially near the edges of the redistributed regions. The Chevron’s stacked configuration also enhanced interfacial area compared to the single SFE. The I.O. SFE demonstrated a more complex redistribution pattern due to its rotating and swapping mechanisms. The side material streams (initially in the shell region) were rotated and partially integrated into the core sections, indicating higher mixing potential in the starting configurations. This was particularly evident in the stacked and rotated configurations, where additional shear and rotational effects disrupted the initial concentric geometry, leading to intermingling of the core and shell materials. While the I.O. SFE maintained the core–shell structure overall, localized mixing at transition points and along rotated material boundaries was more pronounced than in the Chevron SFE.

When using polymer clay to prototype, there are key differences to consider between the behavior of polymers, such as APA, LCP, and polymer clay. Polymer clay consists of fine filler particles (i.e., polyvinyl chloride or plastisol) suspended in a plasticizer, creating a particulate suspension with a semi-solid, putty-like consistency, and lacking true molecular entanglements [9,10]. In contrast, polymers are composed of long-chain molecules whose viscoelastic behavior is governed by shear rate, molecular weight, entanglements, and segmental relaxation dynamics [35,36,37]. Polymer clay also cannot mimic flow-induced behaviors such as molecular alignment, strain hardening, or crystallization. Real polymers, under shear or elongational flow, develop chain alignment along the flow direction, resulting in highly anisotropic mechanical properties [38,39].

The polymer clay extrusion not only validated geometric assumptions within the simulation framework, but also helped identify the root causes of discrepancies observed in the polymer coextrusion simulations. This contrast suggests that one of the sources of the mismatch lies in the material behavior of the polymer system, including interfacial tension, viscosity contrast, and temperature dependence, none of which were factors in the polymer clay tests. Another key factor is die wall texture. The polymer clay dies were fabricated via Fused Deposition Modeling (FDM) 3D printing, which introduces surface roughness that is absent in simulations, which assume perfectly smooth, no-slip boundaries. Studies have shown that wall texture can affect wall slip and flow uniformity, especially in shear-thinning or paste-like systems. For example, Larrazabal et al. [40] found that increasing the die roughness altered the critical shear stress and modified local flow in polyolefins, while Piau et al. demonstrated that slippery or textured surfaces can change extrudate shape and reduce surface distortions [41]. These effects were present in both polymer clay and polymer extrusion, but not captured in simulation.

Strain hardening in semicrystalline polymers arises from strain-induced molecular orientation and crystallization during cooling, which is an effect absent in polymer clay [39]. This viscoelastic response, rather than the plastic-yield behavior of clay, allows polymers to recover elastically after deformation, enabling energy dissipation and toughness under load [42]. Without entanglements, polymer clay flows as a yield stress fluid, undergoing irreversible deformation with minimal recovery [10,36]. Additionally, polymers undergo post-processing transformations, cooling and solidification, which establish the microstructure and mechanical properties. Crystallization kinetics in APA and LCP influence the final morphology, toughness, and thermal stability. These factors are nonexistent in polymer clay [43]. While polymer clay offers a visual approximation of material displacement through the SFEs, it cannot replicate anisotropic reinforcement, interfacial dynamics, or processing-induced microstructure [38,44,45]. Consequently, while polymer clay provided a useful visualization of bulk material displacement, it could not replicate the complex interfacial dynamics or flow-induced anisotropy expected in the polymer system, leading to significant deviations between the prototype and final extrudate cross-sections [46,47].

### 3.3. Microscopy

#### Polymers

**Figure 12:**
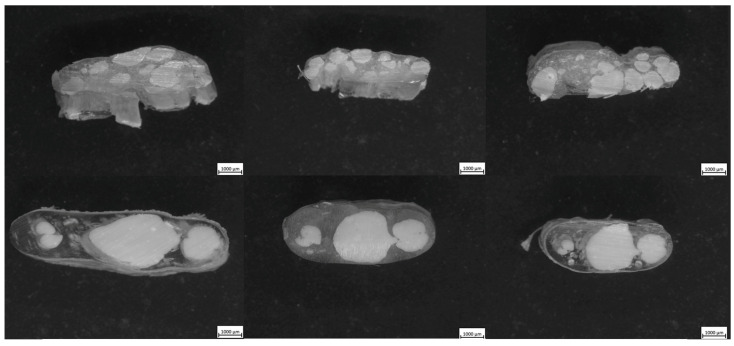
Resulting microscopy of cross-sections transverse to flow for I.O. SFE (**top**) and Inv. Chevron SFE (**bottom**). In both cases, the LCP remained concentrated in the core while the APA occupied the outer regions. The Chevron geometry introduced sharper transitions, while the I.O. allowed minor LCP intrusions near the edges.

The microscopy of the coextruded samples revealed how the final composites compared with the predictions from simulation, shown in Figure 12. For the Chevron SFE, the diamond shape in the center of the flow path introduced sharp transitions and higher differences in shear rates through the direction of flow. The LCP remains in the core of the composite due to the initial core–shell starting point, and the APA resists the shear deformation despite being located at the die wall, one of the regions of higher shear in the die. In the I.O. SFE, the higher-viscosity APA still remains near the outer regions of the composite, but minor intrusions of LCP are allowed due to the nature of the die. At the processing temperature, the viscosity mismatch between APA and LCP is relatively high, which likely contributes to the observed minor intrusions and deviations from simulation predictions. Lowering the processing conditions would reduce the viscosity difference, promoting more uniform flow and closer agreement with simulated distributions. Additionally, while the use of compatibilizers could improve interfacial adhesion and reduce localized boundary disturbances, they are not expected to completely eliminate the redistribution effects caused by the inherent viscosity difference and complex SFE geometry.

### 3.4. Structural

#### Polymers

**Figure 13:**
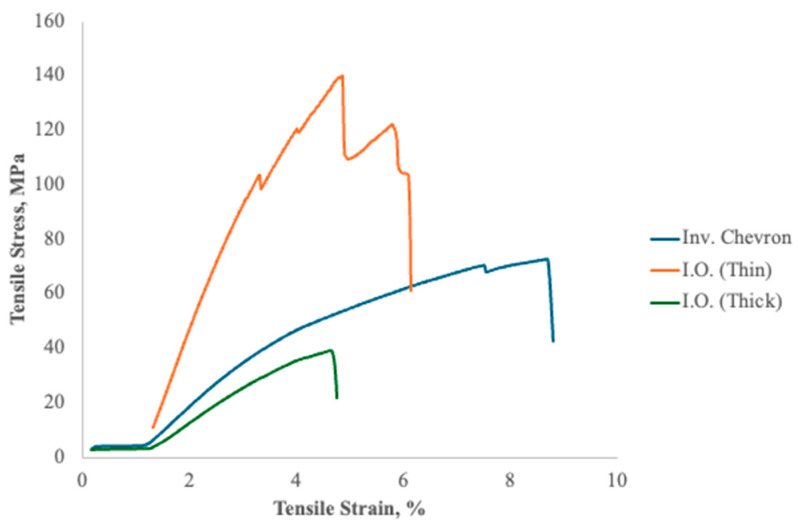
Resulting representative stress–strain plots for Inv. Chevron and I.O. with two different draw ratios. Chevron samples fractured abruptly at low strains with minimal plastic deformation, reflecting a more brittle failure. In contrast, the I.O. samples showed both a higher modulus and stress at break, with more extensive elastic regions, indicating improved stiffness and toughness through LCP alignment.

The representative stress–strain behavior, shown in Figure 13, of the coextruded composites revealed insights into the mechanical performance of the materials under tensile loading, with significant differences arising between the Chevron and I.O. configurations. For the Chevron samples, the stress–strain curves displayed limited ductility, characterized by abrupt fractures at relatively low strains. These fractures initiated from stress concentrations caused by the uneven material distribution and geometric irregularities inherent in the SFE design. Thus, the Chevron samples exhibited brittle failure, fracturing abruptly at low strains with minimal plastic deformation.

In contrast, the stress–strain curves for the I.O. samples exhibited marked improvements, particularly in the tensile modulus and stress at break. These samples showed more extensive linear elastic regions, indicative of higher stiffness achieved through the alignment of the LCP phase during the drawing process. The I.O. configuration enabled smoother transitions in material flow, reducing stress concentrations and promoting uniform molecular alignment. Consequently, these samples resisted higher tensile stresses before failure, underscoring the superior mechanical synergy achieved with this SFE design.

However, no consistent trend in the strain at break was observed. This lack of correlation is primarily attributed to variation between samples and the presence of weak interfacial adhesion between the immiscible materials. While an increased draw ratio promoted molecular alignment and improved the stiffness and strength, the interfaces between the materials did not provide sufficient load transfer during deformation. As a result, local defects and weak bonding sites governed the failure process, producing wider variation in the strain-at-break values.

**Figure 14:**
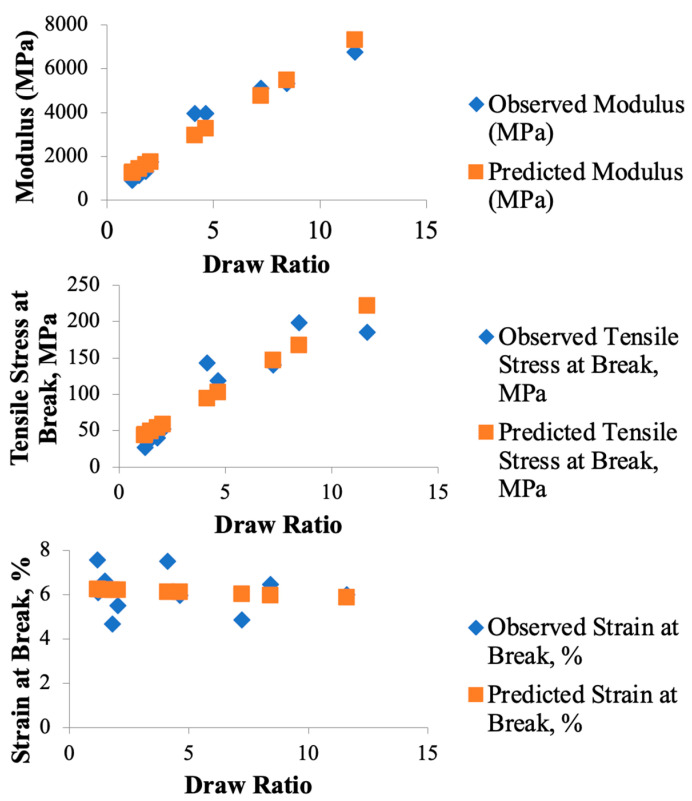
Scatter plots of observed vs. predicted regression results for modulus, stress at break, and strain at break of I.O. SFE samples. The plots highlight the strong dependence of stiffness and strength of the samples on the draw ratio, in contrast with the draw ratio having no significant correlation for strain at break.

Multiple regression analysis modeled the effect of draw on the modulus, tensile stress at break, and strain to failure, with the respective results in Table 3, Table 4, and Table 5. Scatter plots of the observed and predicted results from the linear regression analysis are shown in Figure 14. The modulus of the I.O. samples demonstrated a strong positive relationship with the draw ratio (adjusted R^2^ = 0.9387), reflecting the role of molecular alignment in enhancing stiffness. As the draw ratio increased, the rod-like molecules in the LCP phase aligned more effectively along the extrusion direction, resulting in stiffer composites. The effect of the draw ratio on stress at break (adjusted R^2^ = 0.838) was also significant, although less pronounced than for modulus. This improvement is attributed to the enhanced interfacial adhesion between the LCP and APA phases, facilitated by the smoother material distribution in the I.O. configuration. Strain at break exhibited no significant correlation with draw ratio (adjusted R^2^ = −0.106), likely due to significant variation in elongation across samples. These statistical results highlight a limitation of the composite system: while increased alignment improves stiffness and strength, it also reduces ductility. The brittle nature of the aligned LCP phase dominates the composite’s overall mechanical behavior at high draw ratios, limiting its ability to absorb energy before failure. The fracture behavior observed in microscopy was also mirrored in the stress–strain curves. The abrupt, catastrophic failures in the Chevron samples aligned with the steep drops in stress seen in their curves, indicative of brittle failure modes. In contrast, the more gradual failure in the I.O. samples was evident in the smoother and more extended stress–strain responses, where the material exhibited more energy absorption before failure. The combination of regression analysis and stress–strain behavior emphasizes the I.O. configuration’s ability to optimize mechanical properties, making it the preferred SFE design for applications demanding high stiffness and tensile strength. Interfacial instabilities might be mitigated through advanced compatibilization strategies, such as the incorporation of linear or graft multiblock copolymers, which have proven highly effective at stabilizing interfaces in immiscible polymer systems [48].

## 4. Conclusions

This study demonstrates the potential of coextruded architected polymer composites to achieve tailored mechanical properties by integrating the selected material system of coextruded LCP and APA, SFE designs, and processing conditions. A combination of simulations, polymer clay modeling, and mechanical testing was used to understand the interactions between flow behavior of different materials and the geometry of the SFEs.

The rheological behavior of APA, LCP, and the polymer clay was essential for interpreting flow behavior and validating simulations with experimental results. The Cross–WLF model described the shear- and temperature-dependent viscosity of the APA/LCP system, while the BCY model captured the pseudoplastic behavior of the polymer clay. Simulations of polymer clay allowed the effect of geometry to be isolated from rheological contrast, providing a useful baseline for evaluating SFE performance. In particular, the Chevron configuration produced interface evolution that closely matched the polymer prototypes, while the I.O. configuration showed slightly more deviation, especially in the polymer system. These discrepancies were attributed to factors not captured by the idealized simulation, including die surface texture, wall slip, and the large viscosity mismatches between the LCP and APA phases.

The significant differences in cross-sections in the polymer coextrusion from the simulation predictions were consistent with interfacial flow instabilities commonly reported in systems with strong rheological differences. These results reinforce the importance of combining physical prototyping with simulation to assess geometric effects independently of material complexities. The polymer clay model, in particular, served as a control that helped distinguish between geometry-driven and material-driven discrepancies in flow behavior. Simulations also revealed the importance of matching the rheological properties of core and shell materials for effective shaping. The low-viscosity, shear-thinning behavior of LCP enabled molecular alignment under extensional flow, which contributed to enhanced stiffness and strength along the extrusion direction.

Experimental characterization also confirmed that the draw ratio was a key parameter influencing composite mechanical performance. Regression analysis showed that the modulus had the strongest correlation with the draw ratio, followed by stress at break, while strain at break remained largely unaffected, highlighting a tradeoff between reinforcement and ductility.

Despite the advances achieved with the I.O. configuration and optimized draw ratios, this study also identified challenges associated with anisotropy and brittleness. While alignment improves stiffness and strength along the flow direction, the transverse properties remain limited, increasing susceptibility to transverse fractures. Future work should explore a different design approach to ensure a more accurate result between the predicted SFE cross-section during the validation process and also during coextrusion with polymeric materials.

## Figures and Tables

**Table 1:** Comparison of mechanical and physical properties between Vectra A950 (LCP) and Trogamid CX7323 (APA), where the LCP has a higher tensile modulus, yield stress, melt temperature, and density, while the APA shows a higher yield strain.

Property	Vectra A950	Trogamid CX7323
Tensile Modulus (MPa)	7800	1400
Yield Stress (MPa)	148	60
Yield Strain (%)	5.7	8
Melting Temperature (°C)	280	250
Density (kg/m^3^)	1400	1020

**Table 2:** Process settings used for both SFEs, including zone temperatures and screw speed. The table also provides die and SFE geometry specifications.

**Process Settings**		
	Chevron	I.O.
Die temperature, °C	295	280
Zone 1, °C	290	280
Zone 2, °C	280	280
Zone 3, °C	270	270
Screw speed, RPM	12	12
**Die and SFE Geometry Specifications**		
Die plate thickness, mm	10	
Die orifice, mm^2^	19.1	
SFE retainer plate thickness, mm	13	
SFE thickness, mm	13	
Chevron SFE orifice, mm^2^	118.26	
I.O.SFE orifice, mm^2^	138.69	

**Table 3:** Regression coefficients for modulus of I.O. SFE samples. The analysis showed a strong positive relationship with the draw ratio (adjusted R^2^ of 0.9387), indicating increased stiffness due to enhanced molecular alignment.

	Coefficients	Standard Error	t Stat	*p*-Value
Intercept	559.520	273.789	2.043	0.0752
Draw Ratio	579.043	49.139	11.783	2.462 × 10^−6^

**Table 4:** Regression coefficients for tensile stress at break of I.O. SFE samples. Stress at break increased with the draw ratio (adjusted R^2^ of 0.838), reflecting an improvement in load transfer due to material distribution.

	Coefficients	Standard Error	t Stat	*p*-Value
Intercept	23.192	13.728	1.689	0.130
Draw Ratio	17.002	2.464	6.900	1.245 × 10^−4^

**Table 5:** Regression coefficients for tensile strain at break of I.O. SFE samples. No significant correlation with the draw ratio was observed (adjusted R^2^ of −0.106), consistent with high variability in elongation and the brittle nature of LCP.

	Coefficients	Standard Error	t Stat	*p*-Value
Intercept	6.262	0.522	11.985	2.164 × 10^−6^
Draw Ratio	−0.034	0.094	−0.366	0.724

**Figure A1:**
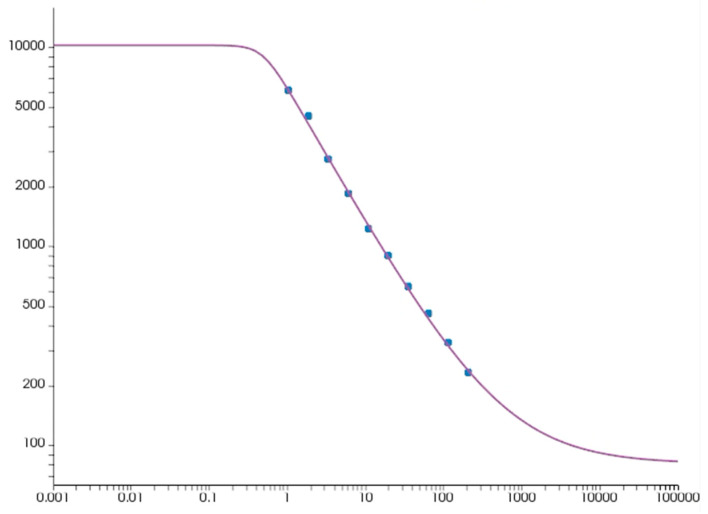
Rheological data fitted with the BCY model for polymer clay.

## Data Availability

The original contributions presented in this study are included in the article. Further inquiries can be directed to the corresponding author(s).

## Abbreviations

The following abbreviations are used in this manuscript:

APA	Amorphous Polyamide
BCY	Bird–Carreau–Yasuda
FDM	Fused Deposition Modeling
L/D	Length to Diameter
LCP	Liquid Crystalline Polymer
LME	Layer-Multiplying Element
PA	Polyamide
SFE	Shape-Forming Element
WLF	Williams–Landel–Ferry

## Appendix A. Clay Characterization and Simulation Details

**Table A1:** Bird–Carreau–Yasuda coefficients.

Coefficients	Polymer Clay
$\eta_{\infty }$	8.0858 × 10^−1^
$\eta_{0}$	1.0269 × 10^4^
λ	2.0831 × 10^−1^
a	3.6913
n	3.1016 × 10^−1^

**Table A2:** Simulation settings utilized in the evaluation of the SFEs with polymer clay.

**Simulation Settings**	
Problem type	Generalized Newtonian Isothermal Flow
Flow rate, m^3^/s	2 × 10^−11^
Core material	Polymer Clay
Shell material	Polymer Clay
Core material radius, mm	4.37
Element size, mm	0.25
Adaptive sizing	On
Number of elements, Chevron	214,424
Number of elements, I.O.	380,995
Mesh resolution	3
**Boundary Conditions**	
Die wall	Zero shear velocity (no slip condition)
Inlets	2 specified, core and shell inlet
Adaptive meshing at material interface	None
Outlets	1 specified, slot die geometry
Convergence strategy	Multiple material flows

## Appendix B. Polymer Characterization and Simulation Details

**Table A3:** Cross–WLF coefficients for Vectra A950 and Trogamid CX7323.

Coefficients	Vectra A950	Trogamid CX7323
n	0.3083	0.1897
τ, Pa	22005.8	493322
D1, Pa⋅s	6.55 × 10^10^	3.0509 × 10^12^
D2, K	473.15	323.15
D3, K⋅Pa^−1^	0	0
A1	30.041	26.999
A2, K	51.6	51.6

**Table A4:** Simulation settings utilized in the evaluation of the SFEs with polymers.

**Simulation Settings**	
Problem type	Generalized Newtonian Isothermal Flow
Flow rate, m^3^/s	1 × 10^−6^
Core material	Vectra A950
Shell material	Trogamid CX7323
Core material radius, mm	4.37
Element size, mm	0.25
Adaptive sizing	On
Number of elements, Chevron	751,706
Number of elements, I.O.	1,104,751
Mesh resolution	4
**Boundary Conditions**	
Die wall	Zero shear velocity (no slip condition)
Inlets	2 specified, core and shell inlet
Adaptive meshing at material interface	None
Outlets	1 specified, slot die geometry
Convergence strategy	Multiple material flows
